# Ebola Virus Glycoprotein Needs an Additional Trigger, beyond Proteolytic Priming for Membrane Fusion

**DOI:** 10.1371/journal.pntd.0001395

**Published:** 2011-11-15

**Authors:** Shridhar Bale, Tong Liu, Sheng Li, Yuhao Wang, Dafna Abelson, Marnie Fusco, Virgil L. Woods, Erica Ollmann Saphire

**Affiliations:** 1 Department of Immunology and Microbial Science, The Scripps Research Institute, La Jolla, California, United States of America; 2 Department of Medicine, University of California San Diego, La Jolla, California, United States of America; 3 The Skaggs Institute for Chemical Biology, The Scripps Research Institute, La Jolla, California, United States of America; University of Texas Medical Branch at Galveston, United States of America

## Abstract

**Background:**

*Ebolavirus* belongs to the family *filoviridae* and causes severe hemorrhagic fever in humans with 50–90% lethality. Detailed understanding of how the viruses attach to and enter new host cells is critical to development of medical interventions. The virus displays a trimeric glycoprotein (GP_1,2_) on its surface that is solely responsible for membrane attachment, virus internalization and fusion. GP_1,2_ is expressed as a single peptide and is cleaved by furin in the host cells to yield two disulphide-linked fragments termed GP1 and GP2 that remain associated in a GP_1,2_ trimeric, viral surface spike. After entry into host endosomes, GP_1,2_ is enzymatically cleaved by endosomal cathepsins B and L, a necessary step in infection. However, the functional effects of the cleavage on the glycoprotein are unknown.

**Principal Findings:**

We demonstrate by antibody binding and Hydrogen-Deuterium Exchange Mass Spectrometry (DXMS) of glycoproteins from two different *ebolaviruses* that although enzymatic priming of GP_1,2_ is required for fusion, the priming itself does not initiate the required conformational changes in the ectodomain of GP_1,2_. Further, ELISA binding data of primed GP_1,2_ to conformational antibody KZ52 suggests that the low pH inside the endosomes also does not trigger dissociation of GP1 from GP2 to effect membrane fusion.

**Significance:**

The results reveal that the *ebolavirus* GP_1,2_ ectodomain remains in the prefusion conformation upon enzymatic cleavage in low pH and removal of the glycan cap. The results also suggest that an additional endosomal trigger is necessary to induce the conformational changes in GP_1,2_ and effect fusion. Identification of this trigger will provide further mechanistic insights into *ebolavirus* infection.

## Author Summary

*Ebolavirus* causes often fatal hemorrhagic fever in humans and nonhuman primates. During infection, the virus is internalized into the low pH endosomes prior to the delivery of viral RNA to the infected cell. Cleavage by endosomal cathepsins of the heavily glycosylated mucin-like domain and glycan cap from the *ebolavirus* surface glycoprotein GP_1,2_ is an essential step in infection. The effect of cleavage and the low pH of the endosomes on the conformation of GP_1,2_ is as yet unknown. To investigate the effect of priming, we cleaved the mucin-like domain and glycan cap of *Zaire ebolavirus* (ZEBOV) GP_1,2_ with thermolysin and engineered a mutant of *Sudan ebolavirus* (SEBOV) GP_1,2_ that is cleaved with furin. We demonstrate by DXMS and antibody binding studies that cleavage of the mucin domain and glycan cap and incubation at low pH are insufficient to trigger the conformational changes of GP_1,2_ that effect fusion. Unraveling the trigger that leads to the conformational change of GP_1,2_ to its fusogenic form will enhance the understanding of *ebolavirus* infection and pinpoint key sites for therapeutic intervention.

## Introduction

*Ebolaviruses* cause severe hemorrhagic fever in humans and non-human primates with 50–90% lethality. No specific vaccines or treatments for *ebolavirus* infection have yet approved for human use [1]–[5]. Among the five different members of the *ebolavirus* genus, *Zaire ebolavirus* (ZEBOV) and *Sudan ebolavirus* (SEBOV) are the most lethal and are the most commonly associated with outbreaks among humans [6]. The virus displays a trimeric glycoprotein (a class I fusion protein) on its surface, termed GP_1,2_, which is solely responsible for attachment and internalization of the virus [7], [8]. The glycoprotein is initially expressed as a single polypeptide that is then cleaved by furin in the producer cell to yield two disulphide-linked subunits termed GP1 and GP2 [9]. Of these, GP1 attaches to target cells while GP2 drives the fusion of viral and host cell membranes for the delivery of viral RNA into the host cells. The GP_1,2_ trimer is extended by heavily glycosylated mucin-like and “glycan cap” regions that are attached to the top of GP1 by a single polypeptide and reach upwards and outwards toward the target cell. The extensive glycocalyx provided by these domains and other glycans on GP1 and GP2 may shield the complex from immune surveillance and/or play an additional role in the natural host reservoir [10].

Numerous studies have revealed that the 450 kDa trimeric GP_1,2_ is further proteolytically cleaved, after entry into target cells, by the endosomal cathepsins L and B [11]–[14]. This trimming operates on the loop formed by residues 190–213 in GP1 and yields a ∼39 kDa fragment containing the N-terminal portion of GP1 (prior to the cleavage site) and all of GP2. Cleavage is thought to expose the receptor-binding region (RBR) on the remaining GP1 core and enhance fusion of viral and cellular membranes [11], [13], [15]. Cathepsins L and B cleave at slightly different sites. Cathepsin L cleaves at residue 201 [15], [16] and is sufficient to remove the mucin-like domain and glycan cap. Cathepsin B deletes additional residues N-terminal to the site of cathepsin L cleavage, removing an additional ∼1 kDa of mass from GP1 [11], [15].

Cleavage by the combination of cathepsins L and B can be functionally mimicked by thermolysin [12], [15]. Thermolysin cleaves GP around residue 190, leaving residues 33–190 of GP1 and all of GP2 [15] that together assemble a ∼39 kDa GP_1,2_ core. Although it is known that these cleavage events are usually required for infection, the structural manifestation of enzymatic cleavage is as yet unclear.

Here we demonstrate by antibody binding and peptide amide hydrogen-deuterium exchange mass spectrometry (DXMS) that priming of the GP_1,2_ ectodomain and endosomal pH themselves are insufficient for triggering the conformational changes necessary for fusion, and that an additional trigger must be required in the infected cell.

## Materials and Methods

### Protein expression and purification

The design of a construct amenable to high-level expression of *Zaire ebolavirus* GP_1,2_ (ZEBOV-GP_1,2_) and KZ52 is described previously [17], [18]. Briefly, ZEBOV-GP_1,2_ has an N-terminal HA tag and comprises residues 33–632 with the mucin and transmembrane regions (residues 313–465 and 633–676) deleted, and a T230V mutation that significantly improves expression yields. The protein was transiently expressed in HEK293T cells and purified using an anti-HA column followed by size exclusion chromatography. ZEBOV-GP_1,2_ is cleaved by thermolysin (75 µg/ml) overnight and the cleaved protein (ZEBOV-GP_1,2CL_) is separated from released fragments by size exclusion chromatography using a Superdex-200 10/300 GL column equilibrated in PBS buffer. To obtain a complex between ZEBOV-GP_1,2CL_ and KZ52, the proteins were mixed in a ratio of 1∶1.5 by weight respectively and incubated on ice for 2 h. The complex was separated from excess KZ52 by size exclusion chromatography (Suppl. Figure S1A).

A construct for *Sudan ebolavirus* GP_1,2_ (SEBOV-GP_1,2_; strain Gulu) was cloned into the pDISPLAY vector and contains a N-terminal HA tag for purification. The construct comprises residues 33–637 with the mucin and transmembrane regions (residues 314–472 and 638–676) deleted. SEBOV-GP_1,2_ is more sensitive to cathepsin and thermolysin cleavage and it is difficult to obtain sufficient yields of cleaved, wild-type SEBOV-GP_1,2_ for DXMS studies. Hence, for production of homogeneous SEBOV-GP_1,2CL_, a furin cleavage site was introduced with the mutation ^205^RKKR^208^ into the 190–213 loop upon which cathepsins and thermolysin act (gift of Paul Bates, University of Pennsylvania). This protein is thus “born” cleaved twice by furin in the producer cell: one furin-cleavage site separates GP1 from GP2, while the second removes the glycan cap from GP1. This “born-cleaved” protein was transiently expressed in HEK293T cells supplemented with a plasmid encoding excess furin (in a glycoprotein-to-furin plasmid ratio of 4∶1 by weight). The expressed protein was purified using an anti-HA column. Further size exclusion chromatography illustrates it is the same size as cleaved wild-type GP_1,2_.

### Effect of pH on KZ52 binding to GP_1,2CL_

Binding of KZ52 to ZEBOV-GP_1,2CL_ under various pH conditions was determined by ELISA. Briefly, aliquots of ZEBOV-GP_1,2CL_ were buffer exchanged to a range of pH (pH 4.2, 4.6, 5.0, 5.4, 6.0, 6.6 and 7.0, each obtained from a 1 M stock solution pH screen (Hampton) using Millipore Ultramax 10 K cutoff centrifugal concentrators [17]), and incubated in 10 mM buffer and 150 mM NaCl at room temperature for 2 h. The proteins were subsequently neutralized in 100 mM Tris-HCl buffer pH 7.5 (note that any conformational changes in GP2 leading to fusion are irreversible). The changes in pH were monitored by litmus paper.

0.25 µg of the protein per well was adsorbed (∼50 µl/well) overnight at 4°C in a 96-well microtiter plate. The wells were washed with PBS containing 0.05% Tween (PBS-Tween) and blocked with 100 µl/well of 3% bovine serum albumin (BSA) in PBS for 1 h at room temperature. The wells were washed (with PBS-Tween) and incubated with 50 µl of the antibody between 0.1 and 1 µg/ml for 1 h, washed again, and incubated with HRP conjugate (1∶2000 dilution in 1% BSA and PBS). The wells were washed thoroughly with PBS-Tween, and bound HRP was detected by incubation with 50 µl/well of 1∶1 solution of TMB substrate (Pierce). The reaction was terminated by addition of 50 µl/well of 2 N H_2_SO_4_. The resulting solutions were analyzed by a UV/Vis spectrophotometer microtiter plate reader at 450 nm.

### DXMS methodology - Optimization of fragmentation conditions

Prior to the exchange experiments, disulfide reduction and protein denaturation conditions were identified that produced an optimal pepsin fragmentation pattern under exchange-quench conditions. It was found that incubation with quench buffer with TCEP and denaturant, followed by dilution of denaturant prior to exposure to pepsin gave the best peptide probe coverage map. For ZEBOV, 1 µl of ZEBOV-GP_1,2_ or ZEBOV-GP_1,2CL_ stock solution (at 5.3 mg/ml and 5.5 mg/ml respectively) was diluted into 1 µl of non-deuterated buffer (8.3 mM Tris, pH 7.5, 150 mM NaCl, in H_2_O) at 0°C. Next, 3 µl of 1 M TCEP, 6.4 M GuHCl, pH 2.85 (quench buffer) was added and the sample was incubated for 5 min on ice. Then 15 µl of 0.8% formic acid (FA), 16.6% glycerol (quench diluent) was added to the denatured sample prior to protease digestion.

For SEBOV, 1 µl of SEBOV-GP_1,2_ stock solution (6.3 mg/ml) was diluted with 7 µl of non-deuterated buffer (8.3 mM Tris, 150 mM NaCl, in H_2_O, pH 7.15) at 0°C and then quenched with 12 µl of quench buffer containing 3.2 M GuHCl, 15 mM TCEP, 0.8% formic acid, 16.6% glycerol (pH 2.4). For SEBOV-GP_1,2CL_ sample, 2 µl of protein (5.1 mg/ml) stock solution was diluted into 3 µl of quench buffer (1 M TCEP, 6.4 M GuHCl, pH 2.85) and the sample was incubated for 5 min on ice. Then 15 µl of quench diluent (0.8% FA, 16.6% glycerol) was added. The samples were then frozen on dry ice and stored at −80°C for further analysis.

In order to demonstrate the ability of our methods to detect differences in deuteration of these proteins, GP1 and GP2 of SEBOV-GP1,2_CL_ were separated from each other by adding 10 mM DTT and heating at 37°C for 30 min. The disulfide-reduced sample was prepared and analyzed in a similar fashion as SEBOV-GP1,2_CL_ for DXMS experiments (Figures S4A–S4C in supplementary information).

All samples were subsequently thawed at 4°C and passed over an AL-20-pepsin column (16 µl bed volume, 30 mg/ml porcine pepsin (Sigma)), at a flow rate of 20 µl/min. The resulting peptides were collected on a C18 trap (Michrom MAGIC C18AQ 0.2×2) and separated using a C18 reversed phase column (Michrom MAGIC C18AQ 0.2×50 3 µm 200 Å) running a linear gradient of 0.046% (v/v) trifluoroacetic acid, 6.4% (v/v) acetonitrile to 0.03% (v/v) trifluoroacetic acid, 38.4% (v/v) acetonitrile over 30 min with column effluent directed into an LCQ mass spectrometer (Thermo-Finnigan LCQ Classic). Data were acquired in both data-dependent MS1∶MS2 mode and MS1 profile mode. SEQUEST software (Thermo Finnigan Inc.) was used to identify the sequence of the peptide ions. DXMS Explorer (Sierra Analytics Inc., Modesto, CA) was used for the analysis of the mass spectra as described previously [19].

### Deuterium exchange experiments

Functional deuteration of ZEBOV glycoproteins were performed by diluting 1 µl of ZEBOV-GP_1,2CL_ or ZEBOV-GP_1,2_ stock solution into 3 µl of D_2_O buffer (8.3 mM Tris, 150 mM NaCl, in D_2_O, pD_READ_ 7.2) at 0°C. At 10 sec and 1000 sec, 6 µl of ice-cold quench buffer (6.4 M GuHCl, 1 M TCEP, pH 2.85) was added, samples incubated on ice for 5 min, 10 µl quench diluent (0.8% FA, 16.6% glycerol) was added, and samples frozen on dry ice. The equilibrium-deuterated back-exchange control samples were prepared by diluting 1 µl of ZEBOV-GP_1,2CL_ or ZEBOV-GP_1,2_ into 3 µl of 1% formic acid in 99.9% D_2_O with incubation overnight at room temperature, cooled to 0°C and mixed with 6 µl quench buffer, incubated for 5 min, supplemented with 10 µl quench diluent, and frozen and further processed as above.

Functional deuteration of SEBOV-GP_1,2_ was performed by first diluting 2 µl of SEBOV-GP_1,2_ with 2 µl of non-deuterated buffer (8.3 mM Tris, 150 mM NaCl, in H_2_O, pH 7.15). At 0°C, 4 µl of D_2_O buffer (8.3 mM Tris, 150 mM NaCl, in D_2_O, pD_READ_ 7.2) was added. At 10 sec and 1000 sec, 12 µl of ice-cold quench buffer (3.2 M GuHCl, 15 mM TCEP, 0.8% formic acid, 16.6% glycerol, pH 2.4) was added. For the SEBOV-GP1,2_CL_ sample, 2 µl of SEBOV-GP_CL_ (5.95 mg/ml) stock solution was diluted into 2 µl of D_2_O buffer (8.3 mM Tris, 150 mM NaCl, in D_2_O, pD_READ_ 7.2) at 0°C. At 10 sec and 1000 sec, 6 µl of ice-cold quench buffer (6.4 M GuHCl, 1 M TCEP, pH 2.85) was added, samples incubated on ice for 5 min, 15 µl quench diluent (0.8% FA, 16.6% glycerol) was added.

The centroids of the isotopic envelopes of nondeuterated, functionally deuterated, and fully deuterated peptides were measured using DXMS Explorer and then converted to corresponding deuteration levels with corrections for back-exchange [20].

### Figure preparation

All figures in the manuscript were generated using Pymol and Adobe photoshop.

## Results and Discussion

### Structure and Neutralizing antibodies of ZEBOV and SEBOV

The crystal structure of *Zaire ebolavirus* GP_1,2_ (ZEBOV-GP_1,2_) has been previously determined in its prefusion form, in complex with an antibody termed KZ52 that was derived from a human survivor of *Zaire ebolavirus* infection [17]. The structure reveals that antibody KZ52 binds a conformational epitope on GP_1,2_ where the GP1 and GP2 subunits meet, and illustrates the specificity of KZ52 for the prefusion state of ZEBOV-GP_1,2_. In addition, we have also determined the crystal structure of the prefusion form of *Sudan ebolavirus* GP_1,2_ (SEBOV-GP_1,2_) bound to a novel neutralizing antibody termed 16F6 [21]. 16F6, like KZ52, recognizes a conformational epitope on the prefusion form of SEBOV-GP_1,2_ that bridges the two subunits together. The loop of GP_1,2_ upon which both endosomal cathepsins and thermolysin act (residues 190–213) is mobile and disordered in both structures, and is located at the outer (“side”) surface of the trimeric GP_1,2_.

In these prefusion GP_1,2_ structures, each GP2 in the trimer wraps around its corresponding GP1 in a metastable state. The GP1s form a “clamp” on the metastable prefusion conformation of GP2 and prevent it from springing irreversibly into its fusion-active and more stable six-helix bundle conformation, which has also been crystallized [22], [23]. In order for fusion to occur, GP2 must unwrap from each GP1 in the prefusion complex and rearrange to yield the six-helix bundle conformation of GP2 alone.

### Priming GP_1,2_

To investigate the effects of enzymatic cleavage of GP_1,2_, we digested ZEBOV-GP_1,2_ with thermolysin and purified the resulting protein (ZEBOV-GP_1,2CL_) by size exclusion chromatography. Note that the ZEBOV-GP_1,2_ used for the studies has a deletion of the mucin-like domain to enhance protein expression. Thermolysin functionally mimics cleavage by endosomal cathepsins, but operates at physiological pH, while cathepsins require low pH (∼5.5) for cleavage. Use of thermolysin allows us to decouple the effects of enzymatic cleavage from the effects of low pH. The elution profile of thermolysin-cleaved ZEBOV-GP_1,2CL_ suggests that ZEBOV-GP_1,2_ remains trimeric after cleavage (Suppl. Figure S1A).

SEBOV-GP_1,2_ is quite sensitive to proteolysis and cleavage by either cathepsins L/B or thermolysin results in significant degradation, making it difficult to purify sufficient material for DXMS studies. In order to analyze the effects of enzymatic cleavage of SEBOV-GP_1,2_, we engineered a version of SEBOV-GP_1,2_ with an additional furin site at residues 206–210 in the loop of GP1 that is operated on by cathepsins and thermolysin (the wild-type furin-cleavage site that separates GP1 and GP2 also remains in this construct). The furin-engineered SEBOV-GP_1,2CL_, is stable, is released from the expression host already deleted of its glycan cap and mucin-like domain and structurally mimics enzymatically cleaved SEBOV-GP_1,2_ (Suppl. Figure S1B). Like ZEBOV-GP_1,2CL_, SEBOV-GP_1,2CL_ also exists as a trimer.

### Analysis of conformational changes by DXMS

DXMS is able to measure the ability of deuterium in solvent water to exchange with hydrogens that are covalently bound to peptide amide nitrogen atoms in a protein [24]. To determine if cleavage of GP_1,2_ results in conformational change, we analyzed uncleaved ZEBOV/SEBOV-GP_1,2_ and cleaved ZEBOV/SEBOV-GP_1,2CL_ proteins by DXMS, obtaining 49%/68% and 68%/83% coverage, respectively. Peptide regions throughout the glycoproteins were analyzed. Peptides covalently linked to glycans of heterogeneous mass are not accessible for measurement with currently employed DXMS methods, and therefore result in small gaps in the sequence coverage. Note that enzymatic cleavage removes nearly all N-linked glycans from GP1 and hence greater sequence coverage is obtained for cleaved GP (68%/83%) than uncleaved GP (49%/68%).

For ZEBOV-GP_1,2_ the peptide fragmentation analysis confirms that thermolysin cleaves after the aromatic residues in the disordered loop ^190^KKDFFSS^196^ and deletes the glycan cap (as observed by Dube et. al. [15]). The deuteration levels of ZEBOV-GP_1,2_ and SEBOV-GP_1,2_ (measured over a time of 10–1000 sec) are consistent with the respective crystal structures (PDB code 3CSY and 3S88) in that peptide fragments that are buried or that have amide hydrogen atoms involved in hydrogen bonding show low levels of deuteration.

**Figure 1:**
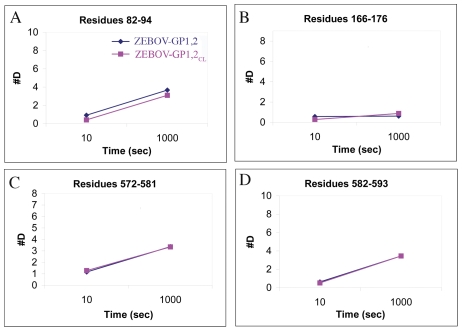
Representative deuteration of peptide fragments of ZEBOV-GP_1,2_. Plots of deuteration of peptides of ZEBOV-GP_1,2_ (shown in dark blue) and ZEBOV-GP_1,2CL_ (shown in magenta) over a time period of 10–1000 sec. Representative peptides are taken from residues adjacent to the glycan cap of GP1 (Panel A: residues 82–94), the base subdomain core region of GP1 (Panel B: residues 166–176), a partially exposed region N-terminal of the trimeric interface of GP2 (Panel C: residues 572–581) and the trimeric interface of GP2 (Panel D: residues 582–593). Deuteration plots of the detected peptides of entire cleaved and uncleaved GP_1,2_ are illustrated in Suppl. Figures S2A–S3C.

**Figure 2:**
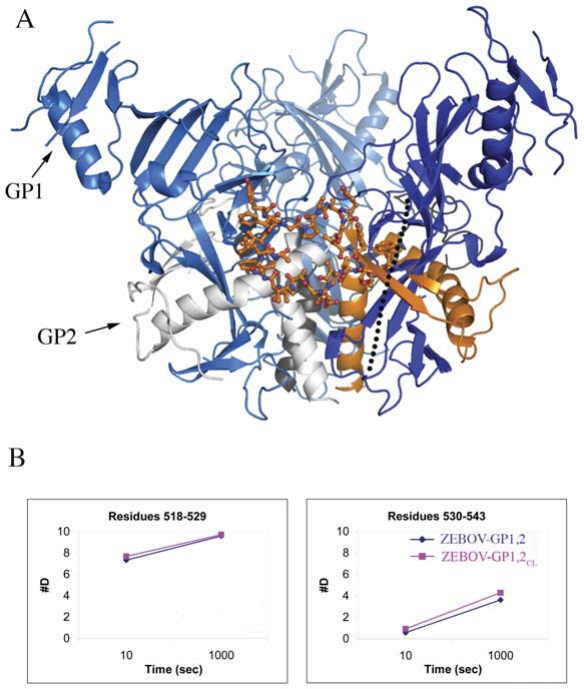
The fusion loop of ZEBOV-GP_1,2_. (A) Cartoon representation of the fusion loop of GP2 (shown in ball-and-stick representation with carbon atoms colored orange). GP1 subunits of the 3-fold related protomers are shown in different shades of blue. One of the GP2 subunits is shown in orange, and the other two are shown in grey. The crystallographically disordered loop that is cleaved by cathepsin L/B is shown as a dotted line. (B) Deuteration plots of the residues in the fusion loop in ZEBOV-GP_1,2_ (shown in dark blue) and ZEBOV-GP_1,2CL_ (shown in magenta).

Comparison of uncleaved GP_1,2_ with cleaved GP_1,2CL_, for both ZEBOV and SEBOV, reveals that no significant changes in deuteration occur upon cleavage for any measured peptide spanning the whole of GP_1,2_, observed over a 10–1000 sec time scale (Figure 1 and S2A–S3C in supplementary information). The deuteration pattern of the residues in the fusion loop (residues 520–540 of GP2) is also identical between the cleaved and uncleaved proteins (Figure 2). The unwinding of the fusion loop is a required early step in the springing of GP_1,2CL_ to the post-fusion form. Alteration in the deuteration pattern of the fusion loop could indicate the beginning of unwinding of GP1,2_CL_, but is not observed after enzymatic cleavage.

**Figure 3:**
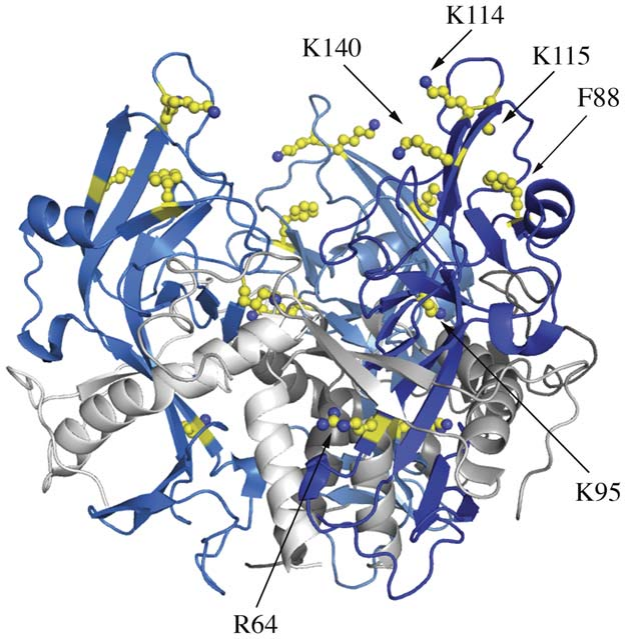
Residues important for attachment. Ribbon representation of a model of the cleaved ZEBOV-GP_1,2CL_ trimer with residues R64, F88, K95, K114, K115 and K140, which have been identified by mutagenesis as important for attachment, shown in ball-and-stick with carbon atoms colored yellow. GP1 subunits are shown in different shades of blue and the GP2 subunits are shown in different shades of grey. Peptides containing residues R64, F88, K95, K114 and K115 do not undergo measurable conformational change upon priming of ZEBOV-GP_1,2_.

In ZEBOV, residues R64, F88, K95, K114, K115, and K140 are critical for binding and are thought to comprise a part of the receptor-binding region [15], [25]. Peptides containing the residues R64, F88, K95, K114, and K115 are equally accessible by solvent before and after cleavage and removal of the glycan cap (Figure 3), indicating that no major conformational changes occur at these sites upon cleavage. Note that peptides containing residue K140 are not detected in DXMS of ZEBOV/SEBOV GP_1,2_ or GP_1,2CL_, and so it is not possible to compare deuteration levels of K140. Of this set of residues, F88, K114, K115 and K140 are inward of the glycan cap and solvent-accessible, and could potentially interact with the receptor. Residues R64 (K64 in SEBOV) and K95 are buried and probably have auxillary roles in receptor binding. Alternately, a conformational change could occur upon receptor binding that brings R64 and K95 into direct contact with the receptor.

DXMS thus suggests that no residues in ZEBOV/SEBOV-GP_1,2CL_ dramatically change conformation when the mucin-like domain and glycan cap are released. These results also suggest that the glycan cap, itself, does not significantly occlude access by solvent to this site. The glycan cap, however, could block steric access by a protein molecule and receptor access would likely be enhanced by enzymatic removal of the glycan cap.

**Figure 4:**
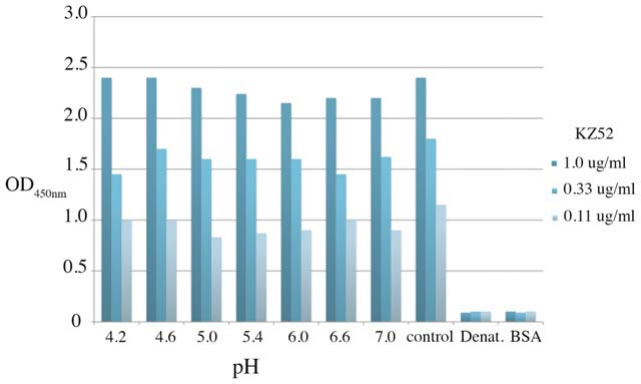
Binding of KZ52 to ZEBOV-GP_1,2CL_ incubated at endosomal pH. Plot of KZ52 binding at 1.0, 0.33, and 0.11 µg/ml (blue, light blue and grey, respectively) to ZEBOV-GP_1,2CL_ in ELISA. Bovine serum albumin (BSA) and denatured ZEBOV-GP1,2_CL_ (Denat.) were used as negative controls. Control ZEBOV-GP_1,2CL_ was maintained at pH 7.5.

The epitope of the human neutralizing antibody KZ52 bridges GP1 and GP2 and KZ52 only binds when the subunits are assembled in their prefusion conformation. Conformational changes in GP2, such as those required for fusion, would likely abrogate KZ52 binding. Hence, binding studies of KZ52 to ZEBOV-GP_1,2CL_ provide additional insights into the structure of ZEBOV- GP_1,2CL_. By both size exclusion chromatography and ELISA, we find that KZ52 binds well to cleaved, trimeric ZEBOV-GP_1,2CL_ (See Figure 4 and Suppl. Figure S1A). An equivalent, GP1/GP2-bridging, prefusion-specific antibody, termed 16F6 [21], that recognizes SEBOV-GP_1,2_ also forms a stable complex with SEBOV-GP_1,2CL_. The binding of the prefusion-specific conformational antibodies KZ52 and 16F6 indicates that the GP_1,2CL_ ectodomain from both viruses remains in its pre-fusion state upon thermolysin or furin cleavage of the 190–213 loop. In addition, successful binding of KZ52 to cathepsin L-cleaved ZEBOV-GP_1,2CL_ with a K_D_ of 1.5 nM has been recently reported by Hood et al. using surface plasmon resonance [16]. By contrast, Shedlock et al. [26] find that KZ52 does not neutralize cathepsin L-cleaved ZEBOV-GP_1,2_ that has been pseudotyped onto a viral surface (binding not directly measured in these studies). Hence, it seems that some as-yet-undetermined differences exist between ectodomain GP and viral-surface GP upon cathepsin L cleavage that do not occur upon thermolysin cleavage.

### Effect of pH on conformation of primed GP_1,2_

The endosomal compartments have an acidic pH ranging from ∼5.9–6.0 in the early endosome to ∼5.0–5.5 in the late endosome and the role of this low pH in triggering irreversible conformational changes leading to fusion has been speculated. To investigate the effect of pH on the conformation of ZEBOV-GP_1,2CL_, we monitored binding of KZ52 to acid pH-treated ZEBOV-GP_1,2CL_ by ELISA (Figure 4). Bovine serum albumin (BSA), and reduced and denatured GP_1,2_ were used as negative controls. Indeed, KZ52 binding is unaffected by incubation of ZEBOV-GP_1,2CL_ in endosomal pH, suggesting that pH alone does not cause rearrangement of ZEBOV-GP_1,2CL_ from the pre-fusion state.

### Conclusions

**Figure 5:**
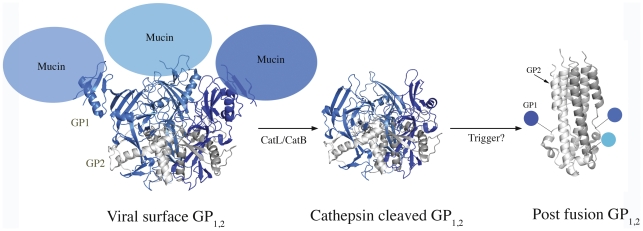
Changing structures of ZEBOV-GP_1,2_. Cartoon representations of GP_1,2_ in its viral surface form (PDB: 3CSY), putative receptor-binding form and post-fusion form (PDB: 2EBO). GP1s and GP2s are colored in different shades of blue and grey, respectively. The mucin-like domains were deleted from ZEBOV-GP_1,2_ for crystallization and have been modeled here as not-to-scale balloons. It is currently unclear if GP1 remains attached to GP2 during the conformational changes that lead to fusion. The transmembrane regions at the bottom of GP_1,2_ are not illustrated.

The DXMS and ELISA binding studies together suggest that the priming of GP_1,2_ of *ebolaviruses* and the low pH in which priming occurs, are themselves, insufficient for triggering the conformational changes required for fusion. An additional trigger such as binding of the receptor to cleaved GP or the action of another cellular factor thus appear to be essential for fusion (Figure 5).

The requirement of enzymatic cleavage for *ebolavirus* GPs may instead serve different, non-exclusive purposes. Cleavage might simply expose the receptor-binding site for binding to an endosomal receptor. Further, removal of the glycan cap and the heavily glycosylated mucin domain (∼75 kDa of protein and carbohydrate) could facilitate membrane fusion by reducing steric barriers to GP2 rearrangement and membrane association. Alternatively, cleavage of the residue 190–213 loop that covers the outside of the fusion loop may remove a flexible tether that anchors the fusion loop in place on the outside of the prefusion trimer. The enzymatic cleavage step of *ebolavirus* GP_1,2_ could indeed be required for one or all of these reasons, but the specific trigger of *ebolavirus* fusion remains to be identified.

## Supporting Information

Figure S1 **Purification of ZEBOV-GP_1,2CL_ and SEBOV-GP_1,2CL_.** (A) Elution profiles of unbound ZEBOV-GP_1,2CL_ (blue) and the complex of ZEBOV-GP_1,2CL_ with Fab KZ52 (red) from Superdex-200 10/300 GL size exclusion chromatography. Bovine serum albumin, a contaminant removed by size exclusion, is abbreviated as BSA. Inset - SDS-PAGE analysis of purified ZEBOV-GP_1,2CL_. (B) SDS-PAGE analysis of purified, “born cleaved” SEBOV-GP_1,2CL_. Precision Plus Protein standards (Biorad cat #161-0374) were used as the molecular weight markers.
(TIF)

Figure S2 **DXMS data for ZEBOV-GP_1,2_ and ZEBOV-GP_1,2CL_.** Number of deuterons vs. time plots of ZEBOV-GP_1,2_ (dark blue) and ZEBOV-GP_1,2CL_ (magenta) for the various fragments (Panels A, B, and C) obtained in DXMS.
(TIF)

Figure S3 **DXMS data for SEBOV-GP_1,2_ and SEBOV-GP_1,2CL_.** Number of deuterons vs. time plots of SEBOV-GP_1,2_ (dark blue) and SEBOV-GP_1,2CL_ (magenta) for the various fragments (Panels A, B, and C) obtained in DXMS.
(TIF)

Figure S4 **DXMS data for native and denatured SEBOV-GP_1,2_.** Number of deuterons vs. time plots of SEBOV-GP_1,2_ (dark blue) and reduced SEBOV-GP_1,2CL_ (magenta) for the various fragments (Panels A, B, and C) obtained in DXMS. Note a significant change in deuteration of GP1 peptides upon reduction of the GP_1,2_ complex.
(TIF)
