# Supplementary figures and images for: Ebola Virus Glycoprotein Needs an Additional Trigger, beyond Proteolytic Priming for Membrane Fusion
Source: PLoS Negl Trop Dis. 2011 Nov 15;5(11):e1395. doi: 10.1371/journal.pntd.0001395 (PMC3216919; doi:10.1371/journal.pntd.0001395)

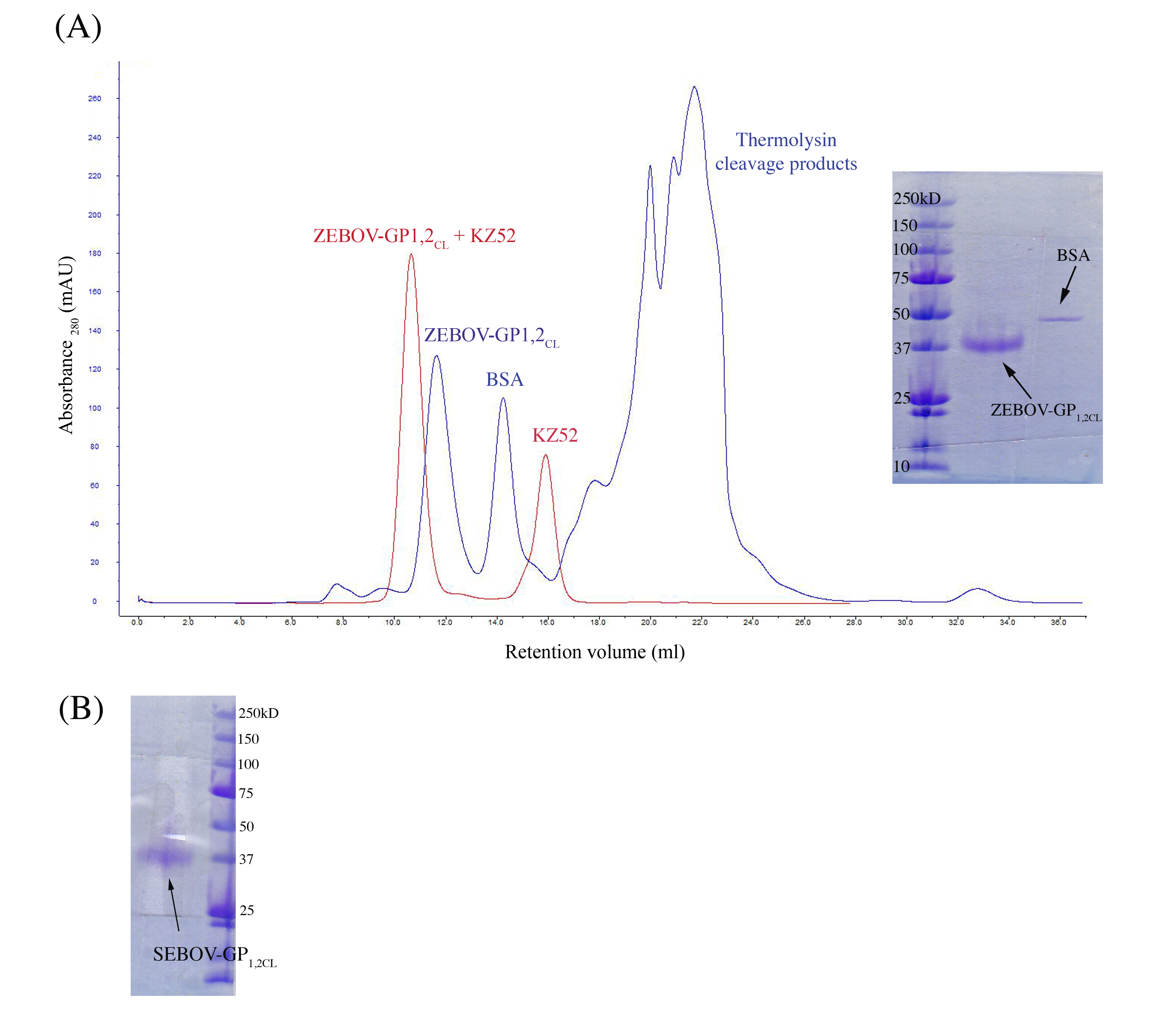

Supplement: Figure S1 — Purification of ZEBOV-GP1,2CL and SEBOV-GP1,2CL. (A) Elution profiles of unbound ZEBOV-GP1,2CL (blue) and the complex of ZEBOV-GP1,2CL with Fab KZ52 (red) from Superdex-200 10/300 GL size exclusion chromatography. Bovine serum albumin, a contaminant removed by size exclusion, is abbreviated as BSA. Inset - SDS-PAGE analysis of purified ZEBOV-GP1,2CL. (B) SDS-PAGE analysis of purified, “born cleaved” SEBOV-GP1,2CL. Precision Plus Protein standards (Biorad cat #161-0374) were used as the molecular weight markers. (TIF) [file pntd.0001395.s001.tif]

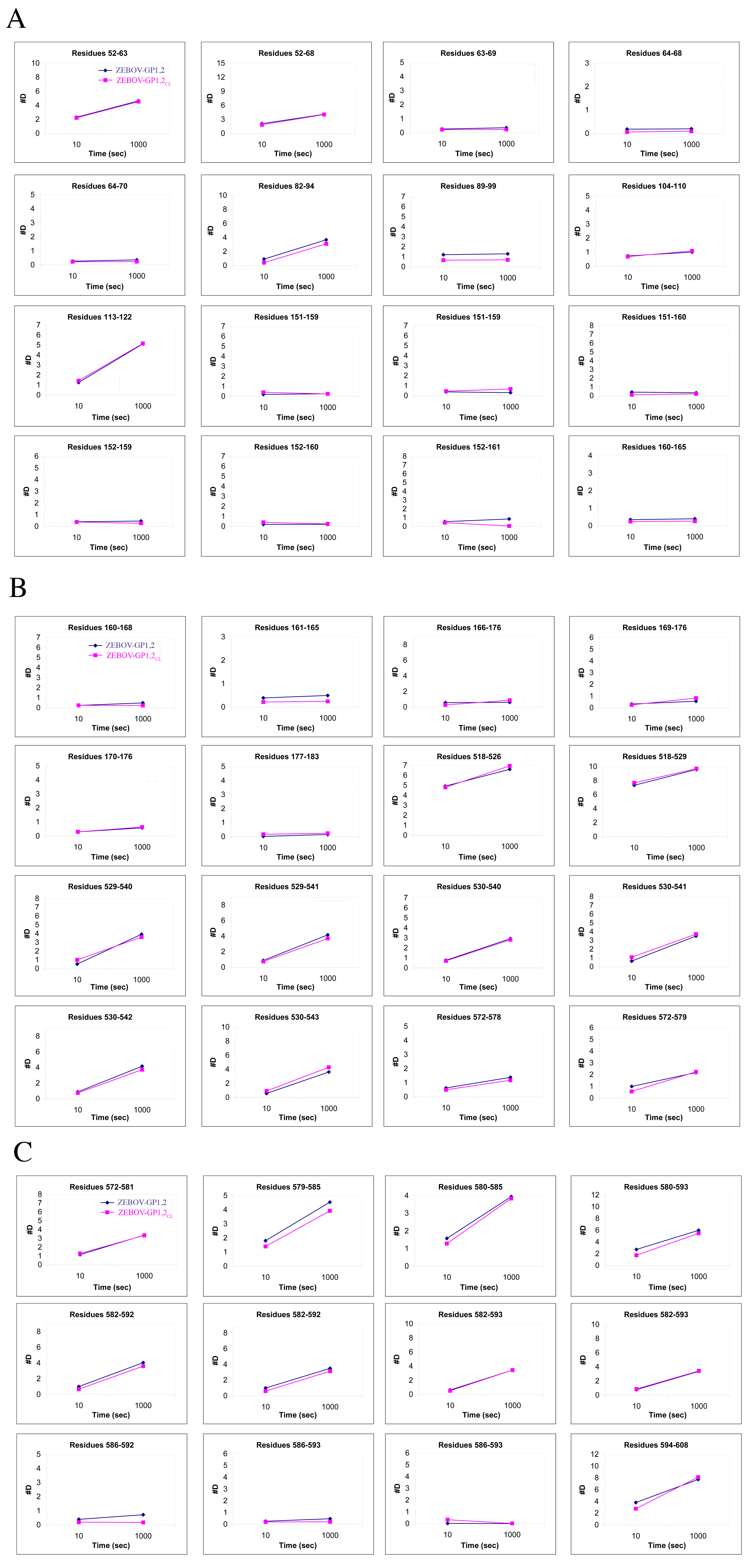

Supplement: Figure S2 — DXMS data for ZEBOV-GP1,2 and ZEBOV-GP1,2CL. Number of deuterons vs. time plots of ZEBOV-GP1,2 (dark blue) and ZEBOV-GP1,2CL (magenta) for the various fragments (Panels A, B, and C) obtained in DXMS. (TIF) [file pntd.0001395.s002.tif]

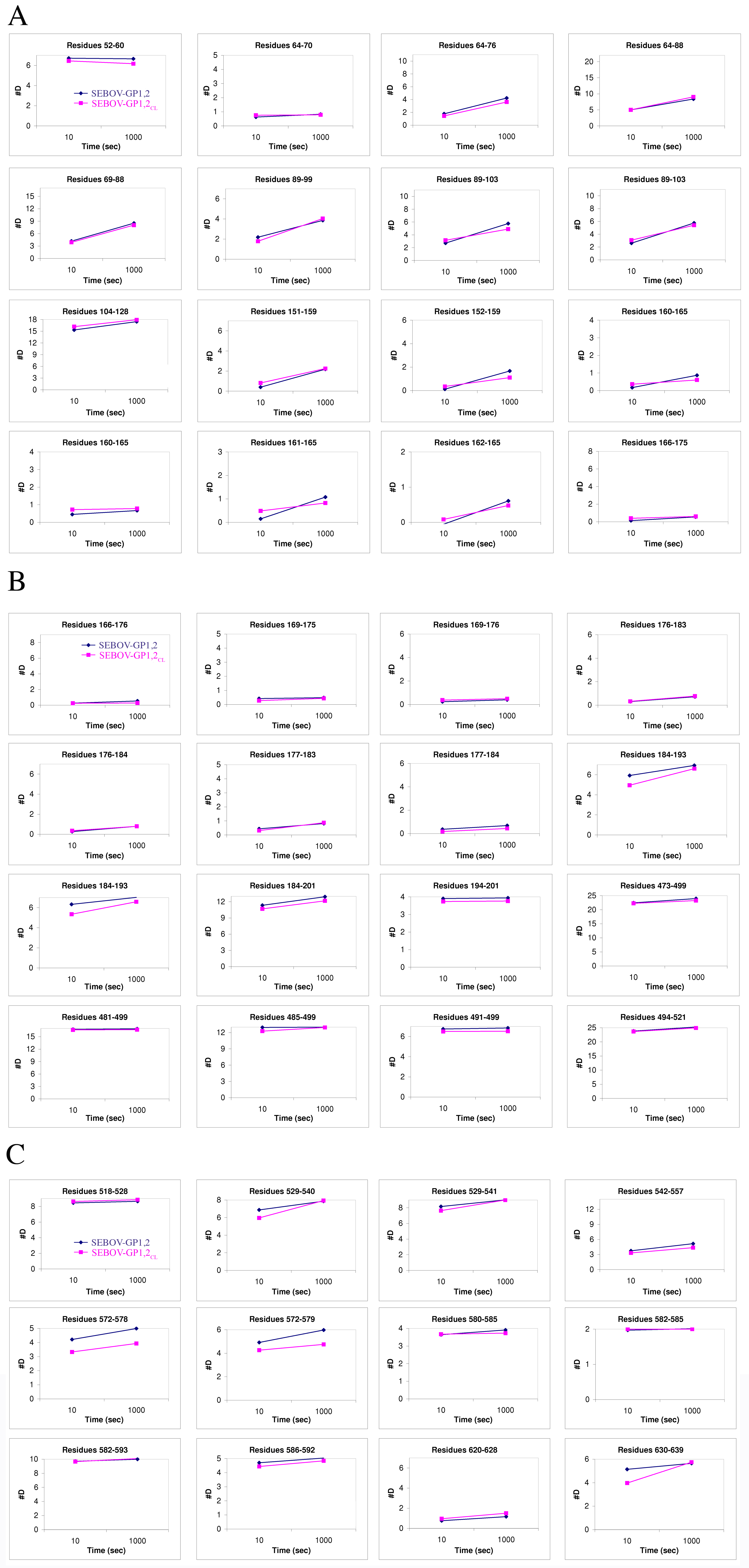

Supplement: Figure S3 — DXMS data for SEBOV-GP1,2 and SEBOV-GP1,2CL. Number of deuterons vs. time plots of SEBOV-GP1,2 (dark blue) and SEBOV-GP1,2CL (magenta) for the various fragments (Panels A, B, and C) obtained in DXMS. (TIF) [file pntd.0001395.s003.tif]

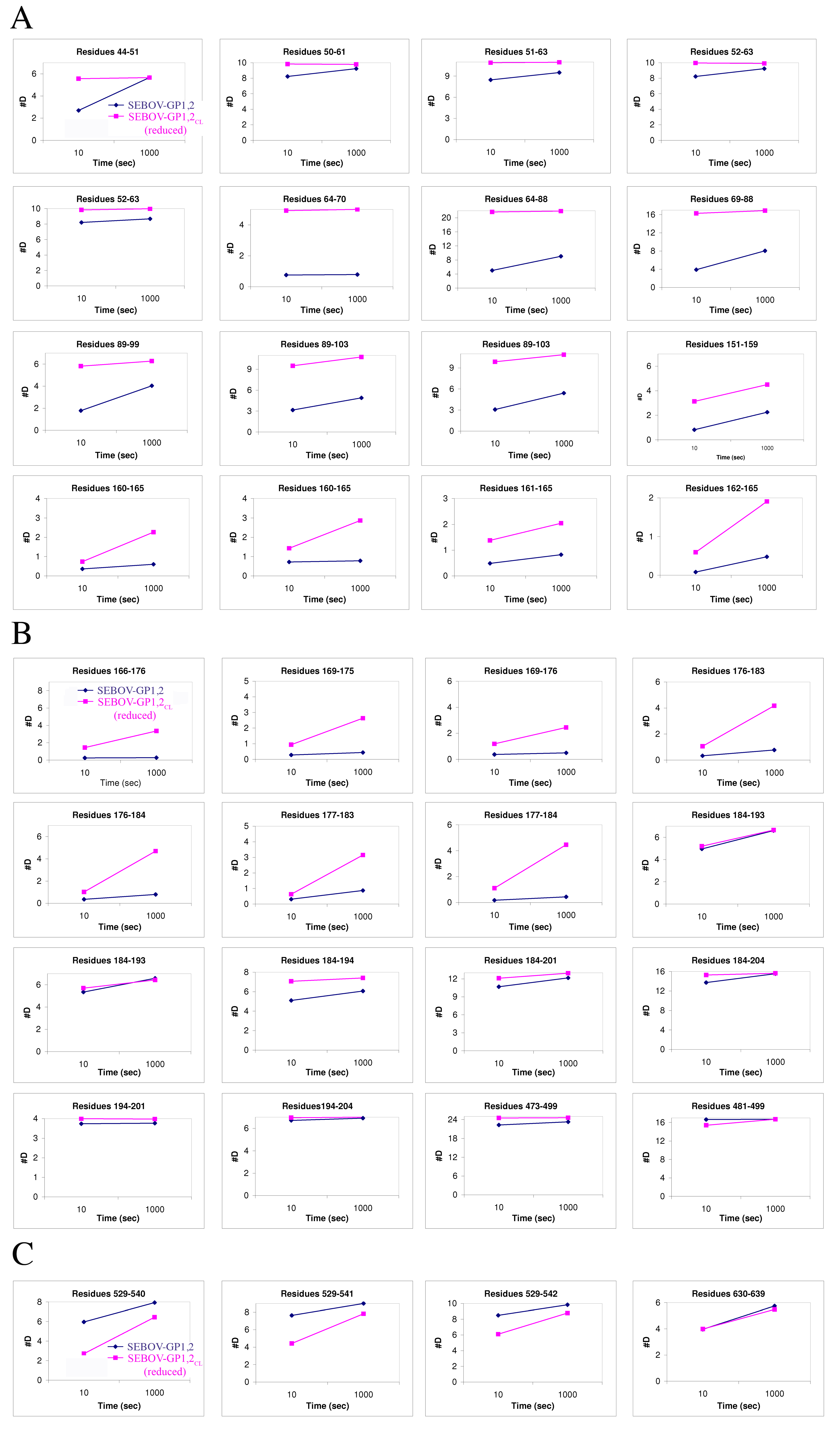

Supplement: Figure S4 — DXMS data for native and denatured SEBOV-GP1,2. Number of deuterons vs. time plots of SEBOV-GP1,2 (dark blue) and reduced SEBOV-GP1,2CL (magenta) for the various fragments (Panels A, B, and C) obtained in DXMS. Note a significant change in deuteration of GP1 peptides upon reduction of the GP1,2 complex. (TIF) [file pntd.0001395.s004.tif]
